# Report of a Case of Typhoid Fever, Showing Range of Temperature from Beginning to the End of the Case

**Published:** 1870-02

**Authors:** T. C. Murphy

**Affiliations:** Green Valley, Ill.


Article VI. — *Report of a Case of Typhoid Fever, Showing the Range of Temperature from the Beginning to the End of the Case.* By T. C. Murphy, Green Valley, Ill.

It will be necessary for me to give a sketch of the residence, mode of living, etc., of the patient, Samuel W., aged 30, occupation farmer. Mr. W.'s residence is situated in a small grove of scrub oak. On the south of the house is a large swamp ; this swamp is covered with water during the spring and part of the summer months, furnishing abundance of malaria — in fact, it might be called the home of ague, the residence being situated so near to the swamp that pure air must be a stranger to the family. Mr. W., being a tenant on the farm, to save time, dug a well at the edge of the swamp. Into this well flowed the washing from the barn-yard, the surface water of the swamp making it a mixture of strength — in fact, it was not fit for a human being to drink. Being at the residence of Mr. W., during the month of June, 1869, I told him to have a well dug on high ground, so as to obtain pure water. He said he had not the time to spare, and he would get along with such as he had. I told him if he did not dig a well he had better hire a physician by the year. During the month of July, Mrs. W. was taken sick; she had the remittent fever. I treated the case according to the indications. She got along well and passed from my care. I saw no more of the family until August 10. Mr. W. called at my office. He wanted medicine. He remarked, "My wife is having chills; she don't seem to be getting along very well. I am all right." A glance showed me such was not the case. He was pale and anæmic — in fact, he looked like a small traveling hospital. I ordered him quinia and iron; and again, "Please get a well dug." He went on his way rejoicing.

We now come to the facts in the case proper. I was called to see Mr. W., August 21. Found him in bed; had a severe chill at 10 A.M.; had fever at the time of my visit; temperature 105° F. Ordered quinia sulph., grs. 3, every three hours, in absence of fever. I could not see the case on next morning. When I did call, I was informed by Mrs. W. that my patient was better — to which I could not agree. I found my patient hard to arouse, unconscious of what was going on. On being aroused, I could get no satisfactory answers from him. I learned from his wife he had passed no urine since my last visit; picked at the bed-clothes during the night; low, muttering delirium; did not complain of pain, and he must be getting better. Flies were having a picnic over his face undisturbed. The family did not seem in the least alarmed. On examination, I found the bladder contained no urine; as he had passed uo urine for near forty-eight hours, I was anxious for the result. Uræmic poisoning was near at hand. Temperature, 102½°. Diagnosis, typhoid fever.

I paid no attention to the treatment of typhoid fever (so called). The indication was: eliminate, or the case will pass through your digits. I ordered a strong decoction of buchu to be taken through the day. I called again at 9 P.M. Patient had passed no urine yet; is stupid, sweating profusely; sweat has a strong ammoniacal odor. On examination, I found the temperature 103½° F. I passed the catheter, and drew off a small quantity of urine; its smell resembled that of the Chicago river. I now started on a voyage of discovery. I asked if there was a cellar under the house. Yes. I examined the cellar; found it contained a mixture of decayed pumpkins, potatoes, bedsteads, old crinoline, boots, fungi (or toad-stools), *et alii.* The house had been banked up with horse manure during the winter; as it decayed, it found an inlet into the cellar—to make an outlet for human life. Do you wonder that typhoid flourished in such a den? I do not. Where the accumulated filth of years becomes concentrated in a cellar, under a house 16 by 16 feet, what wonder that the poison arising from such a "black hole" should act with such force on the nervous system of the patient, oppressing him nearly to the grave. I told the friends of the patient I could do nothing for the case, in such a house, as typhoid had marked it for its home. I at last got permission to move him to a large, clean house in the vicinity. I secured a room, 16 by 20 feet, where I could obtain fresh air, pure water, light, sunshine, and cleanliness, God's choicest blessings to man. Now, having secured a room to suit me, and procuring elimination by the kidneys, we wish to keep up the action of the eliminating organs—the kidneys, lungs, skin and intestines.

Third day: Tongue is covered with a yellow fur; trembles on being put out of the mouth; is dry and parched; tenderness and gurgling in the illiac region; diarrhœa; low, muttering delirium; temperature 104°. During the night the patient was restless; tried to leave his bed. I ordered the following:

℞ Hyd. submur., - - - - - - - - gr. ij;
Pulv. doveri, - - - - - - - - - - gr. j;
Give one every three hours.

Second—

℞ Spts. ether nitric, - - - - - - - - ℥ j;
Fld. ext. veratrum, - - - - - - - gtt. viij; M.
A teaspoonful three times a day.

Object of the above: The first, to secure elimination by the intestines; conveying out of the system, by the agency of the discharges, the morbid material presumed, with great probability, to contain the specific virus of the disease. The intestinal glands eliminate the poison. The object of the second is clear.

Fourth day, 10 A.M.: Temperature, 104°; pulse, 135. Under the combined influence of pure air, sunshine, light and cleanliness, and the eliminating organs doing duty, the stupor has passed off. Patient answers questions quite well; flies creeping over his face attract attention — in fact, a change is apparent to all. What produced it? Cleanliness, in all its virgin purity. The surface of the patient was sponged with tepid water three times a day. The kidneys secrete a sufficient quantity of urine. Treatment continued.

Sixth day, 7 o'clock, P.M: Temperature 104, pulse 130, bowels moved three or four times during the last twenty-four hours, answers questions well but is soon asleep; passed about xxiv ℥; it contains a large amount of excreta; destruction of tissue is rapid, as shown by the high temperature, and the amount of excreta contained in the urine. Ordered Doveri, grs. v; camphor pulv., grs. ij, every four hours during the night, to allay nervous irritability. Ordered the following during the day:

℞. Acid sulph. arom., - - - - - gts. xx;
Quinia sulph., - - - - - - grs. ss:
Aquæ mentha pip. - - - - - ℥ i;
Given every four hours during the day.

Seventh day, 9 A.M.: Temperature 103; pulse 130; bowels moved twice during the night; rested quite well during the night; tenderness on pressure over the abdomen; will take no nourishment only milk and lime water, in small quantities at a time—he says "I don't want any thing to eat; I am not hungry." And now having secured the elimination of the poison in some degree, we must bear in mind, this man must eat to live; we must supply material to repair the excessive tissue-change that has taken place. The indication now is — obviate the tendency to death; support, feed. As I cannot see my patient every day, I order him wine, beef-tea, egg-nog, milk-punch, in such quantities as I think will be digested and assimilated; in fact, any thing which contains the elements of nutrition. Again feed; don't wait for the call of the typhoid case, or he may starve to death:

℞ Acid sulph. aromat., - - - - - gts. xx;
Quinia sulph., - - - - - - grs. j;
Aquæ mentha pip. - - - - - ℥ i; M.
Every four hours during the day.

Doveri in five-grain doses at night, as often as may be required to secure sleep. I don't care so much for medicine, so-called, as I do that the patient receives proper nourishment. Calomel, jalapa, or any other infernal mixture, never rebuilt the smallest part of a simple cell.

Ninth day, 9 o'clock, A.M.: Temperature, 102°; pulse 115; rests well at night; bowels moved three times during the last twenty-four hours. Ordered treatment continued; surface to be sponged night and morning; bed-clothes must be changed every other day; all discharges must be removed at once, vessels to be rinsed with a solution of carbolic acid.

Eleventh day, 6 o'clock, P.M.: Temperature 102½°; pulse 120; bowels moved very often during the night; discharges mostly bloody, evidently containing sloughs of Peyers patches. Ordered opium in half-grain doses, every three hours, until diarrhœa is restrained; wine and milk-punch at stated intervals during the night.

Twelfth day, 8 o'clock, A.M.: Temperature 101°; pulse 105; has rested some during the night; no blood in the discharges this morning. Complains of being hungry; answers questions quite well. Ordered bread and milk diet, in such quantities as may seem harmless to the good judgment of the nurse; the acid mixture, with quinia, every four hours during the day; opium, 1 gr. at night, repeated every four hours, using only sufficient to secure sleep, taking care not to narcotize the patient.

Fourteenth day, 9 A.M.: Temperature 100°; pulse 98; rests well during the night; no diarrhœa; calls for food quite often; patient evidently thinks he must eat to live. As digestion of food is good, I now begin to withdraw the wine, giving plenty of sweet milk (and I dont take off the cream). Nurse reports remissions of the fever in the evening. Medicine is only a secondary object now; quinine 2 grs., every four hours during the day.

Sixteenth day, 10 A.M.: Temperature 98½°; pulse 84; bowels moved twice a day; complains of no pain; eats well. Treatment continued.

Eighteenth day, 8 A.M.: Temperature 96°; pulse 75; no fever; bowels moved once in the last twenty-four hours. No tenderness in the iliac region; sleeps well during the night; eats like a plow-boy. Treatment, quinia and acid.

Twentieth day, 10 A. M.: Temperature 98°; pulse 80; tongue is cleaning off; patient sits up, looking at his long, bony arms; says he thinks there is room for improvement. Can form no idea how long he has been sick, or what has happened; rests well at night; digestion good and rapid; fever has evidently run its career:

℞ Nitro-muriatic acid, - - - - - - - - gts. xx
Three times a day. To be given in sugar and water.

Ordered the patient to be well fed.

Twenty-second day, 9 A.M.: Temperature 98°; pulse 80; rests well at night; tongue is cleaned off; has had no fever since my last visit; no tenderness on pressure over the abdomen; I consider there is no need to give the patient medicine now, and tell him so.

Now, let us look over the history of the case. I contend that typhoid fever was brought on in this case by filth, for the following reasons: There was not a case of typhoid within ten miles of his residence; there were no other cases of typhoid in his vicinity during the past fall. Of five persons who lived in the house, four contracted typhoid fever; all of the same virulent nature. And I will warrant to get up cases of typhoid fever to order for any one who wishes to make the experiment of living over the accumulated filth of years (during the summer months).

A word in regard to my plan of treatment. It may seem to some that my plan of treatment was simple; I think so too. Had I treated the case according to the name of the disease, the case would have slipped through my digits. Never treat a case according to its name or location, but according to what is the matter; referring, at all times, the treatment of the disease to the cause of the disease and the particular stage of the disease. What were the indications in this case? Eliminate during the first stage; obviate the tendency to death, and furnish material to repair the excessive waste of tissue that is going on. Nature removes the poison by elimination. We are but nature's hod-carriers; we furnish the bricks and mortar, nature repairs the structure. In treatment of typhoid fever, keep in view the grand fact, you cannot make something out of nothing.

